# Central Administration of Hydrogen Sulfide Donor NaHS Reduces Iba1-Positive Cells in the PVN and Attenuates Rodent Angiotensin II Hypertension

**DOI:** 10.3389/fnins.2021.690919

**Published:** 2021-09-13

**Authors:** Basak Donertas Ayaz, Aline C. Oliveira, Wendi L. Malphurs, Ty Redler, Alan Moreira de Araujo, Ravindra K. Sharma, Basar Sirmagul, Jasenka Zubcevic

**Affiliations:** ^1^Department of Physiological Sciences, College of Veterinary Medicine, University of Florida, Gainesville, FL, United States; ^2^Department of Pharmacology, College of Medicine, Eskisehir Osmangazi University, Eskisehir, Turkey; ^3^Department of Physiology and Functional Genomics, College of Medicine, University of Florida, Gainesville, FL, United States; ^4^Department of Pharmacodynamics, College of Pharmacy, University of Florida, Gainesville, FL, United States; ^5^Department of Physiology and Pharmacology, Center for Hypertension and Precision Medicine, College of Medicine, University of Toledo, Toledo, OH, United States

**Keywords:** hydrogen sulfide, hypertension, microglia, neuroinflammation, paraventricular nucleus, microbiota, angiotensin

## Abstract

Hydrogen sulfide (H_2_S) is a gaseous signaling molecule with neuromodulatory, anti-inflammatory, and anti-hypertensive effects. Here, we investigate whether chronic intracerebroventricular (ICV) infusion of sodium hydrosulfide (NaHS), an H_2_S donor, can alleviate angiotensin II (Ang II)–induced hypertension (HTN), improve autonomic function, and impact microglia in the paraventricular nucleus (PVN) of the hypothalamus, a brain region associated with autonomic control of blood pressure (BP) and neuroinflammation in HTN. Chronic delivery of Ang II (200 ng/kg/min, subcutaneous) for 4 weeks produced a typical increase in BP and sympathetic drive and elevated the number of ionized calcium binding adaptor molecule 1–positive (Iba1^+^) cells in the PVN of male, Sprague–Dawley rats. ICV co-infusion of NaHS (at 30 and/or 60 nmol/h) significantly attenuated these effects of Ang II. Ang II also increased the abundance of cecal *Deltaproteobacteria* and *Desulfovibrionales*, among others, which was prevented by ICV NaHS co-infusion at 30 and 60 nmol/h. We observed no differences in circulating H_2_S between the groups. Our results suggest that central H_2_S may alleviate rodent HTN independently from circulating H_2_S via effects on autonomic nervous system and PVN microglia.

## Introduction

Uncontrolled, treatment-resistant hypertension (HTN) develops in part due to aberrant autonomic mechanisms characterized by hyperactivity of the sympathetic nervous system (Tsioufis et al., 2011) and impairment of cardiac vagal modulation (Mancia and Grassi, 2014). The paraventricular nucleus (PVN) of the hypothalamus is an important hub integrating a variety of central and peripheral signals in regulation of autonomic pathways for cardiovascular homeostasis (Badoer, 2001; Coote, 2005). Neuroinflammation of PVN, marked by microglia activation and increased microglial cell number (Shi et al., 2010; Santisteban et al., 2015; Sharma et al., 2019), has been associated with sympathetic over-activity and fluid imbalance in rodent HTN, suggesting that microglia may exert effects on autonomic regulation of blood pressure (BP). Microglia are the resident immune cells of the brain involved in the regular maintenance of the neural environment and pruning of synapses, and aberrant pruning and exacerbated neuroinflammation involving activated microglia are implicated in the various neuropathologies (Paolicelli and Ferretti, 2017; Sakai, 2020; Geloso and D’Ambrosi, 2021). Consequently, significant research to date has attempted to understand the role of microglia in many inflammatory conditions including in HTN.

Hydrogen sulfide (H_2_S) is an endogenous signaling molecule produced by the gut bacteria and the host (Kimura, 2014; Donertas Ayaz and Zubcevic, 2020). Impairment in H_2_S homeostasis has been implicated in development of HTN (Zhong et al., 2003; Yan et al., 2004; Yang et al., 2008; Huang et al., 2015) and studies using H_2_S donors have alluded to a therapeutic potential of H_2_S in HTN. To date, the BP-lowering effects of H_2_S have been primarily attributed to direct vascular effects in various animal models of HTN (d’Emmanuele di Villa Bianca et al., 2015; Xue et al., 2015; Ni et al., 2018; Xiao et al., 2018; Li et al., 2019b; Zhu et al., 2021). However, the central effects of H_2_S cannot be discounted, as H_2_S-producing enzymes are present in the brain (Abe and Kimura, 1996). Moreover, H_2_S is a gaseous molecule that reportedly crosses the blood–brain barrier, which may affect the central nervous system (CNS) directly (Che et al., 2018). However, a few studies to date have investigated central effects of H_2_S in HTN (Streeter et al., 2011; Yu et al., 2015; Liang et al., 2017) while precise mechanisms of central BP-lowering effects of H_2_S remain unknown.

Hydrogen sulfide is a neuromodulator with neuroprotective effects (Zhang and Bian, 2014). It can freely cross the cell membrane to regulate several intracellular signaling processes (Zhang and Bian, 2014). Recent studies showed that H_2_S can attenuate glial-mediated neuroinflammation (Xuan et al., 2012; Lee et al., 2013, 2016), and that the PVN is a site of action of H_2_S (Liang et al., 2017). Treatment with H_2_S donors *in vitro* can decrease pro-inflammatory cytokines in glial cells (Lee et al., 2016), while intraperitoneal injection of sodium hydrosulfide (NaHS), an H_2_S donor, lowers pro-inflammatory cytokines and gliosis in the hippocampus (Xuan et al., 2012). Thus, the first objective of this study was to test whether chronic, intracerebroventricular (ICV) infusion of NaHS may alleviate rodent angiotensin II (Ang II)–induced HTN and reduce microglia cell counts in the PVN. Rodent and human HTN have also been linked with gut dysbiosis (Yang et al., 2015; Li et al., 2017; Yan et al., 2017) and inhibition of microglial activity in the PVN can rebalance the gut microbiota in HTN (Sharma et al., 2019). Thus, we further hypothesized that central NaHS administration may attenuate Ang II-induced gut dysbiosis.

The present study shows that chronic ICV infusion of NaHS attenuated Ang II-induced increase in microglial cells in the PVN, alleviated Ang II-dependent autonomic dysfunction, and reduced BP. Lastly, ICV infusion of NaHS prevented Ang II-dependent elevation in several cecal bacterial taxa, including but not limited to the sulfate-reducing *Desulfovibrionales*. No change in circulating H_2_S levels was observed in any of the treatment groups. Thus, H_2_S may have direct central neuromodulating and neuroimmune effects that are beneficial for cardiovascular homeostasis, and thus presents a viable anti-hypertensive target.

## Materials and Methods

All experimental procedures were approved by the University of Florida Institutional Animal Care and Use Committee and complied with the standards stated in the National Institutes of Health Guide for the Care and Use of Laboratory Animals.

### Animals

Eight-week-old, male, Sprague–Dawley (SD) rats (*n* = 31) were purchased from Charles River Laboratories (United States) and housed individually in a temperature-controlled room (22 ± 1°C) of University of Florida Animal Care Service Facility with a 12:12-h light–dark cycle with food and water *ad libitum*.

### Experimental Procedures

#### NaHS and Angiotensin II Delivery, BP Measurement, and Variability Analysis of Systolic BP and Heart Rate

**FIGURE 1:**
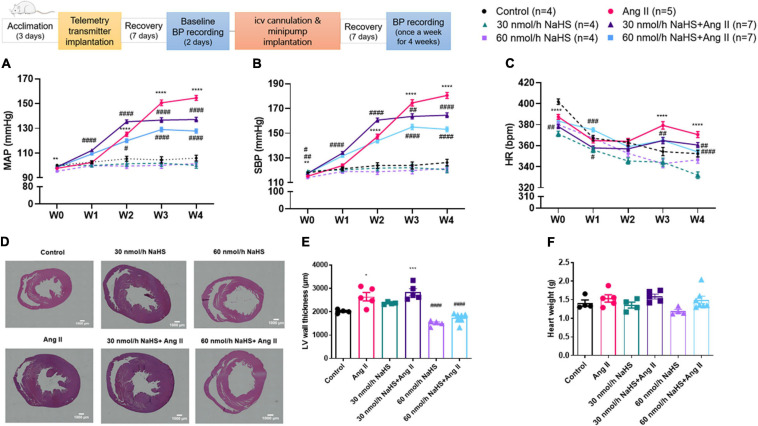
Schematic representation of study design (ICV: intracerebroventricular; BP: blood pressure). Chronic ICV infusion of NaHS attenuated angiotensin (Ang) II–induced increase in **(A)** mean arterial pressure (MAP) and **(B)** systolic blood pressure (SBP) in Sprague–Dawley rats. **(C)** Ang II effect on heart rate (HR). Data was analyzed by Kruskal–Wallis *H* test followed by Tukey’s multiple comparisons test and presented as mean ± SEM. ****p < 0.0001, ***p < 0.001, **p < 0.01, *p < 0.05 control vs. Ang II. ^####^*p* < 0.0001, ^###^*p* < 0.001, ^##^*p* < 0.01, ^#^*p* < 0.05 Ang II vs. NaHS/Ang II-treated rats. In panels **(D–E)**, Ang II-induced left ventricular (LV) hypertrophy as measured by LV thickness was attenuated by 60 nmol/h but not 30 nmol/h NaHS ICV infusion. In panel **(D)**, representative images of H&E stain in ventricular sections of experimental groups (magnification: ×4, scale bar = 1000 μm). In panel **(E)**, LV wall thickness (μm) and **(F)** heart weight (g). Data were analyzed by one-way ANOVA followed by Tukey’s multiple comparisons test and presented as mean ± SEM. ***p < 0.001, *p < 0.05 Control vs. all other groups. ^####^*p* < 0.0001 Ang II vs. all other groups.

A schematic of the timeline used is shown in Figure 1. BP and heart rate (HR) measurements were performed in conscious freely moving rats using radio-telemetry transmitters (HD-S10 model, DSI, Saint Paul, MN, United States, Ponemah software v.6.11). Telemetry transmitters were implanted into the descending abdominal aorta of all rats as previously described (Huetteman and Bogie, 2009). After 7 days of recovery, baseline measurements were taken for 48 h. Then, an ICV brain cannula (ALZET, Durect Corp., Cupertino, CA, United States, Brain Infusion Kit 1 3–5 mm) was implanted into left cerebral ventricle in all rats (coordinates: 1.3 mm anterior–posterior from bregma, 1.50 medial–lateral, and 4.50 dorsal–ventral from skull surface, Paxinos and Watson Rat Brain Atlas) to deliver either NaHS (30 and 60 nmol/h in separate groups of rats; Sigma-Aldrich, United States, CAS Number 207683-19-0) or vehicle phosphate-buffered saline (PBS, 1 × without calcium and magnesium; CORNING Cellgro) for 4 weeks. ICV cannulas were connected to subcutaneous (SC) osmotic minipumps (ALZET, Durect Corp., model 2004, rate of infusion 0.25–0.28 μl per hour). A separate SC osmotic minipump was implanted as well to deliver either Ang II (200 ng/kg/min in normal saline; Bachem, Torrance, CA, United States) or vehicle (normal saline) for 4 weeks. Thus, animals were divided in the following groups: (1) Control (*n* = 4, ICV PBS and SC normal saline); (2) Ang II (*n* = 5, ICV PBS + SC Ang II); (3) 30 nmol/h NaHS (*n* = 4, ICV 30 nmol/h NaHS + SC PBS); (4) 60 nmol/h NaHS (*n* = 4, ICV 60 nmol/h NaHS + SC PBS); (5) 30 nmol/h NaHS + Ang II (*n* = 7, ICV 30 nmol/h NaHS + SC Ang II); (6) 60 nmol/h NaHS + Ang II (*n* = 7, ICV 60 nmol/h NaHS + SC Ang II). After 7 days of recovery, BP and HR measurements were performed once a week for 48 h for 4 weeks. Mean arterial pressure (MAP), systolic blood pressure (SBP), and HR were automatically derived from the Ponemah software (DSI, Ponemah software v.6.11). Variations in SBP and inter-beat interval (IBI) were computed to assess cardiovascular autonomic modulation using Ponemah’s variability analysis module. Variations have been categorized into three frequency bands [very low frequency (VLF) at 0–0.26 Hz, low frequency (LF) at 0.26–0.76 Hz, and high frequency (HF) at 0.76–3.3 Hz (Tayler et al., 2018)] using Fast Fourier transform, and these bands were automatically derived from Ponemah software. To assess variability of SBP, total power of SBP (TP_*SBP*_), an index of global variability, and LF of SBP (LF_*SBP*_), reflecting vasomotor sympathetic modulation (deBoer et al., 1987; Madwed et al., 1989), were derived. For assessment of HR variability, total power of IBI (TP_*IBI*_), VLF of IBI (VLF_*IBI*_), reportedly related to changes in the renin–angiotensin system and thermoregulation (Taylor et al., 1998; Pumprla et al., 2002) and parasympathetic modulation (Taylor et al., 1998), LF of IBI (LF_*IBI*_), which reportedly contains both vagal and cardiac sympathetic components (Shaffer and Ginsberg, 2017), HF of IBI (HF_*IBI*_), reflecting cardiac parasympathetic modulation (Pagani et al., 1986), normalized LF of IBI, representing the relative value of LF_*IBI*_ power component in comparison to the TP, normalized HF of IBI, representing the relative value of HF_*IBI*_ power component in comparison to the TP, and LF/HF of the IBI (LH/HF_*IBI*_), an index of cardiac autonomic balance (Montano et al., 1994), were derived. For these analyses, the averaged SBP and HR variability data was collected for 5 min of every hour for 48 h.

#### Tissue and Cecal Content Collection

At study endpoint, rats were euthanized using 4% isoflurane in 95:5 O_2_/CO_2_. Whole cecal content was collected from all rats and stored at −80°C until analysis. Whole brains were post-fixed in fresh 2% paraformaldehyde (PFA) at 4°C overnight, then transferred to 30% sucrose solution at 4°C for cryoprotection. Brains were then placed in OCT (Tissue-Tek; Sakura Finetek, Torrance, CA, United States) and frozen at −80°C until sectioning. Whole hearts were collected and post-fixed in fresh 2% PFA at 4°C for 48 h, after which they were transferred to 70% ethanol and stored at 4°C until infiltration process.

#### Histology

Before the infiltration process, PFA-fixed hearts were cut transversely into three pieces. On the dorsal aspect of the heart, the first cut was made right below the pulmonary artery, and the second cut was made 5 mm below the first cut. The middle section was then embedded in paraffin using the Tissue-Tek VIP 5 vacuum infiltration processor (Sakura Finetek, United States) and cross-sectioned at 4-μm thickness using a microtome (Accu-Cut SRM 200; Sakura Finetek, United States). Sections with knife trace and/or folding were excluded and one representative slide per rat was stained with H&E using Leica’s ST5020 automated multi-stainer in accordance with the manufacturer’s protocol. Slides were imaged on a Keyence microscope (BZ-X810) under equal conditions and scanned and stitched using × 4 objective. The cross-sectional thickness of the left ventricular (LV) wall was measured from four different areas for one section/rat with ImageJ software, and the results were averaged.

#### Immunohistochemistry

Coronal sections of the hypothalamic PVN (40 μm in thickness; taken at −0.72 mm to −1.92 mm from Bregma, Paxinos and Watson Rat Brain Atlas) were obtained using a cryostat (MICROM HM 505 E; GMI, Ramsey, MN, United States). PVN sections were mounted on superfrost plus slides (Fisher). Slides were stored at −20°C until immunohistochemistry (IHC). Ionized calcium binding adaptor molecule-1 (Iba1) is the most widely used microglia marker of a protein that participates in membrane ruffling and phagocytosis in activated microglia, and elevated level of Iba1 refers to microglia activation (Ohsawa et al., 2004; Jurga et al., 2020). IHC and quantification of Iba1–positive (Iba1^+^) cells in the PVN were performed as previously described (Oliveira et al., 2018; Sharma et al., 2018). Briefly, non-specific binding was blocked with 10% goat serum and Triton X-100 (0.3% in PBS) followed by incubation with rabbit anti-Iba1 primary antibody (1:500; Wako, Richmond, VA, United States) at 4°C overnight. After this, slides were incubated with Alexa Fluor 488–labeled anti-rabbit secondary antibody (1:500; Invitrogen, Carlsbad, CA, United States) for 1 h at room temperature. Slides were cover-slipped using Vectashield mounting media (Vector Labs, Burlingame, CA, United States). Images were taken using a Keyence microscope (BZ-X810) under ×20 objective. For every rat, a stack of 15 PVN images 2 μm apart was compressed to a single image using BZ-X800 Viewer and were analyzed using Fiji-ImageJ cell counter.

#### Analysis of Cecal Bacterial Communities by 16S rRNA Gene Sequencing

Cecal fecal microbial DNA was extracted using MO BIO’s PowerMag Soil DNA Isolation Kit (catalog no. 27100-4-EP) as per manufacturer’s instructions. Bacterial 16S rRNA genes were amplified using primers targeting the V4 region (515F 5′-GTGCCAGCMGCCGCGGTAA-3′ and 806R 5′-GGACTACHVGGGTWTCTAAT-3′), as previously described (Kozich et al., 2013). PCR amplicons were then sequenced in an Illumina MiSeq using the 300-bp paired-end kit (v.3). Sequences were denoised, taxonomically classified using Greengenes (v. 13_8), and clustered into 97% similarity operational taxonomic units (OTUs) with the mothur software (v. 1.39.5) (Schloss et al., 2009). OTUs that were considered putative contaminants were removed if their mean abundance in controls reached or exceeded 25% of their mean abundance in specimens. OTUs were then classified into taxonomic assignments. Assigned taxonomy were organized into an OTU table. Sequencing data are avaliable in the Supplementary Material.

#### Measurement of Plasma H_2_S Levels

Hydrogen sulfide concentration in plasma was assayed spectrophotometrically as described previously (Zhuo et al., 2009). Briefly, 75 μl plasma mixed with 250 μl 1% (w/v) zinc acetate (zinc acetate, anhydrous, 99.9+%; Alfa Aesar, Fisher, United States, CAS Number 557-34-6) and 425 μl distilled water. Then, 20 mM *N*-dimethyl-*p*-phenylenediamine oxalate (Fisher, United States, CAS Number 207683-19-0 CAS 62778-12-5) in 7.2 μM HCI (133 μI) (Fisher, United States, CAS Number 7647-01-0, 7732-18-5) and 30 mM iron trichloride (FeCl_3_) (iron (III) chloride, anhydrous, 98%; Alfa Aesar Fisher, United States, CAS Number 7705-08-0) in 1.2 μM HCI (133 μI) were added and incubated for 10 min at room temperature. Protein in the plasma was removed by adding 250 μl of 50% trichloroacetic acid [Trichloroacetic Acid (Crystalline/Certified); Fisher, United States, CAS Number 76-03-9] to the reaction mixture and pelleted by centrifugation at 12,000 × *g* for 15 min. Then 300 μl of samples were put into each well and absorbance of the solution was read with a spectrophotometer (BioTek Synergy Mx) at 670 nm in a 96-well plate (Fisher). All samples were assayed in duplicate and blank subtracted absorbance values were averaged.

### Data Presentation, Bioinformatics, and Statistical Analysis

Significant differences in BP and variability data were analyzed using IBM SPSS Statistics version 21.0 via Kruskal–Wallis *H* test followed by Tukey’s multiple comparisons test and were presented using GraphPad Prism version 9.0. Significant differences in LV thickness, the number of Iba1^+^ microglial cells, H_2_S plasma assay, Shannon diversity index, and *Firmicutes*/*Bacteroidetes* (F/B) ratio were determined using GraphPad Prism version 9.0 with one-way ANOVA followed by Tukey’s multiple comparisons test or Kruskal–Wallis test where appropriate. Normality of distribution was tested using the Shapiro–Wilk test. A *p* value < 0.05 was considered statistically significant. Data are presented as mean ± SEM. Shannon diversity index is presented as minimum to maximum with a median.

Statistical analyses of cecal bacterial communities were conducted in R. Diversity within a sample is referred to as alpha diversity, and diversity between samples is referred to as beta diversity. Alpha diversity was estimated with the Shannon index on raw OTU abundance tables after filtering out contaminants. The significance of diversity differences was tested with ANOVA. To estimate beta diversity, OTUs occurring with a count of less than 3 in at least 10% of the samples were excluded and then computed using Bray–Curtis indices. Beta diversity, emphasizing differences across samples, was visualized using principal coordinate analysis (PCoA) ordination. Variation in community structure was assessed with permutational multivariate analyses of variance (PERMANOVA) with treatment group as the main fixed factor and using 9,999 permutations for significance testing. A *p* value < 0.05 was considered statistically significant. F/B ratio was calculated by dividing the abundance ratio (percentage of each species in total number of species identified) of *Firmicutes* with that of the *Bacteroidetes*.

Operational taxonomic units were aggregated into each taxonomic rank and plotted the relative abundance (how common or rare an OTU is relative to other OTUs in the same community) of the most abundant ones. Data are presented as mean ± SEM. Linear discriminant analysis effect size (LEfSe) was also employed to identify species that were differentially distributed between different samples^1^. The threshold of the linear discriminant analysis score was 2.0 and alpha value for the Kruskal–Wallis test and Wilcoxon test was 0.05.

## Results

### Central Administration of NaHS Attenuated Angiotensin II–Induced Changes in BP, Heart Rate, and Left Ventricular Wall Thickness

Table 1 summarizes all significant differences between groups and times of treatment. Chronic SC Ang II infusion significantly elevated MAP and SBP starting at week 2 (W2), and these remained elevated through week 4 (W4) of infusion compared with the control groups (Figures 1A,B). Chronic, concomitant ICV infusion of 30 and 60 nmol/h NAHS dose-dependently (Table 1) attenuated Ang II-induced increase in MAP and SBP (Figures 1A,B). HR significantly decreased in all groups over time compared with baseline (W0) (Table 1). However, this time-dependent decrease in HR was significantly dampened by Ang II infusion when compared with control and NaHS/Ang II groups (Figure 1C).

**TABLE 1:** Changes in blood pressure and heart rate in response to subcutaneous Angiotensin II and intracerebroventricular NaHS infusion.

Parameter	Weeks	Control (*n* = 4) (*)	Ang II (*n* = 5) (#)	30 nmol/h NaHS (*n* = 4)	30 nmol/h NaHS + Ang II (*n* = 7) (∧)	60 nmol/h NaHS (*n* = 4)	60 nmol/h NaHS + Ang II (*n* = 7)
**MAP (mmHg)**	0	100.25 ± 0.59	97.72 ± 0.53**	100.03 ± 0.59^##^	98.78 ± 0.45*	95.30 ± 0.59****, ^##^	98.59 ± 0.45*
	1	102.70 ± 1.19^ϕ^	102.44 ± 1.06^ϕϕϕϕ^	99.87 ± 1.19	112.09 ± 0.90^ϕϕϕϕ^, ****, ^####^	99.81 ± 1.19^ϕϕϕϕ^	109.30 ± 0.90^ϕϕϕϕ^, ****, ^####^, ∧
	2	105.39 ± 1.98^ϕ^	125.17 ± 1.77^ϕϕϕϕ^, ****	101.29 ± 1.98^####^	135.32 ± 1.50^ϕϕϕϕ^, ****, ^####^	99.39 ± 1.98^ϕ^, *, ^####^	119.96 ± 1.50^ϕϕϕϕ^, ****, ^#^, ^∧∧∧∧^
	3	104.44 ± 2.54	150.62 ± 2.27^ϕϕϕϕ^, ****	101.80 ± 2.54^####^	136.70 ± 1.92^ϕϕϕϕ^, ****, ^####^	100.14 ± 2.54^####^	129.04 ± 1.92^ϕϕϕϕ^, ****, ^####^, ^∧∧^
	4	105.91 ± 2.35^ϕ^	154.54 ± 2.10^ϕϕϕϕ^, ****	100.06 ± 2.35^####^	137.16 ± 1.78^ϕϕϕϕ^, ****, ^####^	101.38 ± 2.35^ϕ^, ^####^	127.80 ± 1.78^ϕϕϕϕ^, ****, ^####^,^∧∧∧^
**SBP (mmHg)**	0	118.15 ± 0.76	115.24 ± 0.68**	119.67 ± 0.76^####^	117.50 ± 0.57^#^	114.10 ± 0.76***	117.58 ± 0.57^##^
	1	121.27 ± 1.46^ϕ^	123.83 ± 1.30^ϕϕϕϕ^	120.32 ± 1.46	134.02 ± 1.10^ϕϕϕϕ^, ****, ^####^	119.00 ± 1.46^ϕϕϕϕ^, ^#^	131.67 ± 1.10^ϕϕϕϕ^, ****, ^####^
	2	123.95 ± 2.23^ϕϕ^	147.29 ± 1.99^ϕϕϕϕ^, ****	121.76 ± 2.23^####^	160.59 ± 1.68^ϕϕϕϕ^, ****, ^####^	118.91 ± 2.23^ϕ^, ^####^	143.82 ± 1.68^ϕϕϕϕ^, ****, ^∧∧∧∧^
	3	123.92 ± 2.84^ϕ^	174.48 ± 2.54^ϕϕϕϕ^, ****	122.09 ± 2.84^####^	163.64 ± 2.15^ϕϕϕϕ^, ****, ^##^	119.90 ± 2.84^ϕ^, ^####^	154.98 ± 2.15^ϕϕϕϕ^, ****, ^####^, ^∧∧^
	4	126.27 ± 2.65^ϕϕ^	180.53 ± 2.37^ϕϕϕϕ^, ****	120.50 ± 2.65^####^	164.57 ± 2.00^ϕϕϕϕ^, ****, ^####^	121.37 ± 2.65^ϕϕ^, ^####^	153.13 ± 2.00^ϕϕϕϕ^, ****, ^####^, ^∧∧∧∧^
**HR (bpm)**	0	401.86 ± 2.62	387.45 ± 2.35****	371.19 ± 2.62****, ^####^	378.20 ± 1.98****, ^##^	379.99 ± 2.62****, ^#^	383.46 ± 1.98****
	1	367.12 ± 2.58^ϕϕϕϕ^	364.86 ± 2.31^ϕϕϕϕ^	355.97 ± 2.58^ϕϕϕϕ^, **, ^#^	357.91 ± 1.95^ϕϕϕϕ^, **, ^#^	367.14 ± 2.58^ϕϕϕϕ^	375.04 ± 1.95^ϕϕϕ^, *, ^###^, ^∧∧∧∧^
	2	362.90 ± 3.00^ϕϕϕϕ^	364.13 ± 2.69^ϕϕϕϕ^	345.38 ± 3.00^ϕϕϕϕ^, ****, ^####^	356.93 ± 2.27^ϕϕϕϕ^	352.66 ± 3.00^ϕϕϕϕ^, *, ^##^	358.98 ± 2.27^ϕϕϕϕ^
	3	354.31 ± 3.93^ϕϕϕϕ^	379.33 ± 3.52^ϕ^, ****	344.26 ± 3.93^ϕϕϕϕ^, ^####^	364.89 ± 2.97^ϕϕϕϕ^, *, ^##^	342.88 ± 3.93^ϕϕϕϕ^, *, ^####^	364.99 ± 2.97^ϕϕϕϕ^, *, ^##^
	4	352.36 ± 3.23^ϕϕϕϕ^	370.59 ± 2.89^ϕϕϕϕ^, ****	331.86 ± 3.23^ϕϕϕϕ^, ****, ^####^	360.60 ± 2.44^ϕϕϕϕ^, *, ^##^	346.50 ± 3.23^ϕϕϕϕ^, ^####^	354.02 ± 2.44^ϕϕϕϕ^, ^####^

*Ang II, Angiotensin II; MAP, mean arterial pressure; SBP, systolic blood pressure; HR, heart rate.*

*Data was analyzed by Kruskal–Wallis *H* test followed by Tukey’s multiple comparisons test and presented as mean ± SEM.*

*^ϕϕϕϕ^*p* < 0.0001, ^ϕϕϕ^*p* < 0.001, ^ϕϕ^*p* < 0.01, ^ϕ^*p* < 0.05 vs. Baseline.*

****p < 0.0001, ***p < 0.001, **p < 0.01, *p < 0.05 Control vs. all other groups.

*^####^*p* < 0.0001, ^###^*p* < 0.001, ^##^*p* < 0.01, ^#^*p* < 0.05 Ang II vs. NaHS-treated groups.*

*∧∧∧∧*p* < 0.0001, ∧∧∧*p* < 0.001, ∧∧*p* < 0.01, ∧*p* < 0.05 30 nmol/h NaHS/Ang II vs. 60 nmol/h NaHS/Ang II.*

In line with BP increase, Ang II infusion caused a significant increase in the LV wall thickness (Figures 1D,E) compared with the control and 60 nmol/h NaHS groups (Ang II: 2,643 ± 179.6 vs. control: 2,016 ± 40.9 μm, *p* < 0.05 and vs. 60 nmol/h NaHS: 1,500 ± 64.0 μm, 60 nmol/h NaHS/Ang II: 1,728 ± 79.4 μm, *p* < 0.0001). The Ang II-induced LV thickness was attenuated by 60 nmol/h NaHS ICV but not 30 nmol/h NaHS ICV co-treatment (Figures 1D,E, Ang II: 2,643 ± 179.6 vs. 60 nmol/h NaHS/Ang II: 1,728 ± 79.4 μm, *p* < 0.0001). No significant difference was found in heart weights between the groups (Figure 1F).

### Central Effects of NaHS on Angiotensin II–Induced Imbalance in Autonomic Variables

**FIGURE 2:**
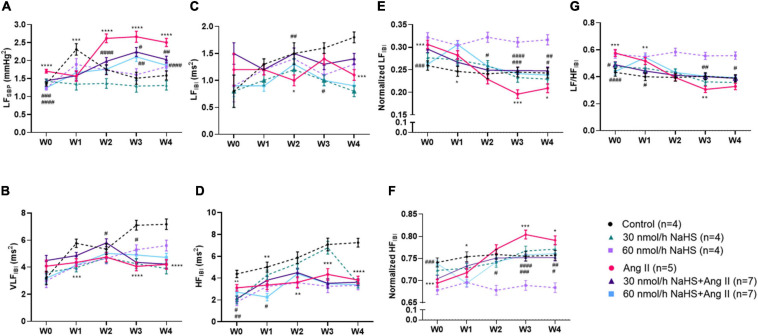
Chronic intracerebroventricular infusion of NaHS attenuates angiotensin (Ang) II–induced autonomic imbalance in Sprague–Dawley rats. Spectral analysis of blood pressure waveform signal was used to derive **(A)** low frequency (LF) of systolic blood pressure (SBP), **(B)** very low frequency (VLF) ratio of inter-beat interval (IBI), **(C)** low frequency (LF) ratio of IBI, **(D)** high frequency (LF) ratio of IBI, **(E)** normalized LF ratio of IBI, **(F)** normalized HF ratio of IBI, and **(G)** LF/HF ratio of IBI. Data were analyzed by Kruskal–Wallis *H* test followed by Tukey’s multiple comparisons test and presented as mean ± SEM. ****p < 0.0001, ***p < 0.001, **p < 0.01, *p < 0.05 control vs. Ang II. ^####^*p* < 0.0001, ^###^*p* < 0.001, ^##^*p* < 0.01, ^#^*p* < 0.05 Ang II vs. NaHS/Ang II-treated rats.

Table 2 summarizes the statistical significance in derived autonomic variables between groups and times of treatment. Spectral analysis of SBP variability showed that Ang II infusion significantly increased TP_*SBP*_ and LF_*SBP*_ starting at W2 compared with control groups, and that ICV NaHS co-treatment significantly attenuated this increase at 60 nmol/h for TP_*SBP*_ and at 30 and 60 nmol/h for LF_*SBP*_ (Table 2 and Figure 2A).

**TABLE 2:** Changes in autonomic variables in response to subcutaneous Angiotensin II and intracerebroventricular NaHS infusion.

Parameter	Weeks	Control (*n* = 4) (*)	Ang II (*n* = 5) (#)	30 nmol/h NaHS (*n* = 4)	30 nmol/h NaHS + Ang II (*n* = 7) (∧)	60 nmol/h NaHS (*n* = 4)	60 nmol/h NaHS + Ang II (*n* = 7)
**LF of SBP (mmHg^2^)**	0	1.35 ± 0.06	1.70 ± 0.05****	1.42 ± 0.06^###^	1.44 ± 0.04^###^	1.24 ± 0.06^####^	1.24 ± 0.04^####^, ^∧∧^
	1	2.31 ± 0.16^ϕϕϕϕ^	1.57 ± 0.14***	1.35 ± 0.16****	1.58 ± 0.12***	1.89 ± 0.16^ϕϕϕϕ^	1.66 ± 0.12^ϕϕϕ^, **
	2	1.75 ± 0.14^ϕϕ^	2.62 ± 0.12^ϕϕϕϕ^, ****	1.37 ± 0.14*, ^####^	1.98 ± 0.10^ϕϕϕϕ^, ^####^	1.74 ± 0.14^ϕϕϕϕ^, ^####^	1.76 ± 0.10^ϕϕϕϕ^, ^####^
	3	1.51 ± 0.17	2.66 ± 0.15^ϕϕϕϕ^, ****	1.29 ± 0.17^####^	2.24 ± 0.13^ϕϕϕϕ^, **, ^#^	1.61 ± 0.17^ϕ^, ^####^	2.10 ± 0.13^ϕϕϕϕ^, **, ^##^
	4	1.59 ± 0.13	2.51 ± 0.11^ϕϕϕϕ^, ****	1.31 ± 0.13^####^	2.03 ± 0.10^ϕϕϕϕ^, **, ^##^	1.83 ± 0.13^ϕϕϕϕ^, ^####^	1.87 ± 0.10^ϕϕϕϕ^, ^####^
**Total Power of SBP**	0	6.22 ± 0.25	6.27 ± 0.23	6.66 ± 0.25	6.99 ± 0.19*, ^#^	4.27 ± 0.25****, ^####^	5.74 ± 0.19^∧∧∧∧^
	1	10.16 ± 0.62^ϕϕϕϕ^	6.84 ± 0.56****	5.85 ± 0.62****	8.45 ± 0.47^ϕϕ^, *, ^#^	7.42 ± 0.62^ϕϕϕϕ^, **	8.27 ± 0.47^ϕϕϕϕ^, *
	2	7.49 ± 0.65	12.03 ± 0.58^ϕϕϕϕ^, ****	5.93 ± 0.65^####^	12.17 ± 0.49^ϕϕϕϕ^, ****	6.12 ± 0.65^ϕϕ^, ^####^	9.89 ± 0.49^ϕϕϕϕ^, **, ^##^, ^∧∧^
	3	7.42 ± 0.75	13.46 ± 0.67^ϕϕϕϕ^, ****	5.82 ± 0.75^####^	11.74 ± 0.56^ϕϕϕϕ^, ****	6.55 ± 0.75^ϕϕ^, ^####^	10.84 ± 0.56^ϕϕϕϕ^, ***, ^##^
	4	7.10 ± 0.60	12.55 ± 0.54^ϕϕϕϕ^, ****	5.63 ± 0.60^####^	11.18 ± 0.45^ϕϕϕϕ^, ****	7.16 ± 0.60^ϕϕϕϕ^, ^####^	9.89 ± 0.45^ϕϕϕϕ^, ***, ^###^, ∧
**VLF of IBI (ms^2^)**	0	3.20 ± 0.50	4.07 ± 0.45	3.60 ± 0.50	4.49 ± 0.38*	2.98 ± 0.50	3.18 ± 0.38∧
	1	5.76 ± 0.32^ϕϕϕϕ^	4.35 ± 0.28***	3.96 ± 0.32****	4.85 ± 0.24*	4.13 ± 0.32^ϕ^, ***	4.14 ± 0.24^ϕ^, ****, ∧
	2	5.33 ± 0.39^ϕϕϕ^	4.75 ± 0.35	4.71 ± 0.39	5.80 ± 0.30^ϕϕ^, ^#^	4.65 ± 0.39^ϕϕ^	5.02 ± 0.30^ϕϕϕϕ^
	3	7.10 ± 0.36^ϕϕϕϕ^	4.06 ± 0.32****	4.25 ± 0.36****	4.35 ± 0.27****	5.29 ± 0.36^ϕϕϕϕ^, ***, ^#^	4.90 ± 0.27^ϕϕϕϕ^, ****, ^#^
	4	7.17 ± 0.40^ϕϕϕϕ^	4.20 ± 0.35****	3.90 ± 0.40****	4.23 ± 0.30****	5.59 ± 0.40^ϕϕϕϕ^, **, ^##^	4.72 ± 0.30^ϕϕϕ^, ****
**LF of IBI (ms^2^)**	0	0.79 ± 0.32	1.20 ± 0.29	0.79 ± 0.32	1.53 ± 0.24	0.85 ± 0.32	0.88 ± 0.24
	1	1.32 ± 0.11	1.16 ± 0.10	0.99 ± 0.11*	1.23 ± 0.08	1.18 ± 0.11	0.93 ± 0.08**, ∧
	2	1.51 ± 0.16	1.01 ± 0.14*	1.22 ± 0.16^#^	1.54 ± 0.12^##^	1.38 ± 0.16	1.27 ± 0.12
	3	1.57 ± 0.13^ϕ^	1.36 ± 0.12	0.95 ± 0.13**	1.34 ± 0.10	1.12 ± 0.13*	1.04 ± 0.10**, ^#^, ∧
	4	1.83 ± 0.15^ϕϕ^	1.13 ± 0.13***	0.82 ± 0.15****	1.42 ± 0.11*	1.28 ± 0.15**	0.90 ± 0.11****, ^∧∧∧^
**HF of IBI (ms^2^)**	0	4.38 ± 0.34	3.10 ± 0.30**	1.97 ± 0.34****, ^#^	2.11 ± 0.25****, ^#^	1.81 ± 0.34****, ^##^	2.71 ± 0.25****, ^##^
	1	5.01 ± 0.46	3.36 ± 0.41**	4.28 ± 0.46^ϕϕϕϕ^	3.81 ± 0.34^ϕϕϕϕ^, *	3.24 ± 0.46^ϕϕ^, **	2.26 ± 0.34****, ^#^, ^∧∧^
	2	5.87 ± 0.53^ϕϕ^	3.61 ± 0.48**	5.36 ± 0.53^ϕϕϕϕ^, ^#^	4.50 ± 0.40^ϕϕϕϕ^, *	3.58 ± 0.53^ϕϕ^, **	4.39 ± 0.40^ϕϕϕϕ^, *
	3	7.08 ± 0.58^ϕϕϕϕ^	4.34 ± 0.52^ϕ^, ***	6.78 ± 0.58^ϕϕϕϕ^, ^##^	3.52 ± 0.44^ϕϕ^, ****	3.25 ± 0.58^ϕϕ^, ****	3.48 ± 0.44****
	4	7.25 ± 0.40^ϕϕϕϕ^	3.82 ± 0.36****	3.50 ± 0.40^ϕϕϕ^, ****	3.63 ± 0.30^ϕϕϕϕ^, ****	3.33 ± 0.40^ϕϕϕ^, ****	3.39 ± 0.30^ϕ^, ****
**LF/HF of IBI**	0	0.43 ± 0.0.03	0.58 ± 0.03***	0.47 ± 0.03^##^	0.49 ± 0.02^#^	0.57 ± 0.03**	0.44 ± 0.02^####^
	1	0.40 ± 0.03	0.52 ± 0.03^ϕ^, **	0.46 ± 0.03	0.44 ± 0.02^ϕ^, ^#^	0.55 ± 0.03***	0.54 ± 0.02^ϕϕϕϕ^, ***, ^∧∧^
	2	0.39 ± 0.03	0.39 ± 0.02^ϕϕϕϕ^	0.43 ± 0.03	0.41 ± 0.02^ϕϕ^	0.58 ± 0.03****, ^####^	0.43 ± 0.02
	3	0.40 ± 0.03	0.31 ± 0.02^ϕϕϕϕ^, **	0.36 ± 0.03^ϕϕϕ^	0.40 ± 0.02^ϕϕϕ^, ^##^	0.56 ± 0.03****, ^####^	0.40 ± 0.02^##^
	4	0.39 ± 0.03	0.33 ± 0.02^ϕϕϕϕ^	0.36 ± 0.03^ϕϕϕ^	0.39 ± 0.02^ϕϕϕϕ^	0.56 ± 0.03****, ^####^	0.39 ± 0.02^#^
**Normalized LF of IBI**	0	0.26 ± 0.01	0.31 ± 0.01***	0.28 ± 0.01*, ^#^	0.30 ± 0.01	0.32 ± 0.01****	0.26 ± 0.01^###^, ^∧∧^
	1	0.25 ± 0.01	0.28 ± 0.01^ϕ^, *	0.27 ± 0.01	0.27 ± 0.01^ϕϕϕ^	0.30 ± 0.01***	0.31 ± 0.01^ϕϕϕϕ^, ****, ^∧∧∧^
	2	0.24 ± 0.01	0.23 ± 0.01^ϕϕϕϕ^	0.26 ± 0.01	0.25 ± 0.01^ϕϕϕϕ^	0.32 ± 0.01****, ^####^	0.26 ± 0.01^#^
	3	0.24 ± 0.01	0.20 ± 0.01^ϕϕϕϕ^, ***	0.23 ± 0.01^ϕϕϕ^, ^#^	0.25 ± 0.01^ϕϕϕϕ^, ^####^	0.31 ± 0.01****, ^####^	0.24 ± 0.01^ϕ^, ^###^
	4	0.24 ± 0.01	0.21 ± 0.01^ϕϕϕϕ^, *	0.23 ± 0.01^ϕϕϕϕ^	0.25 ± 0.01^ϕϕϕϕ^, ^##^	0.32 ± 0.01****, ^####^	0.24 ± 0.01^ϕϕ^, ^#^
**Normalized HF of IBI**	0	0.74 ± 0.01	0.69 ± 0.01***	0.72 ± 0.01^#^	0.70 ± 0.01**	0.68 ± 0.01****	0.74 ± 0.01^###^, ^∧∧^
	1	0.75 ± 0.01	0.72 ± 0.01^ϕ^, *	0.73 ± 0.01	0.73 ± 0.01^ϕϕϕ^	0.70 ± 0.01***	0.69 ± 0.01^ϕϕϕϕ^, ****, ^∧∧∧^
	2	0.76 ± 0.01	0.77 ± 0.01^ϕϕϕϕ^	0.74 ± 0.01	0.75 ± 0.01^ϕϕϕϕ^	0.68 ± 0.01****, ^####^	0.74 ± 0.01^#^
	3	0.76 ± 0.01	0.80 ± 0.01^ϕϕϕϕ^, ***	0.77 ± 0.01^ϕϕϕ^, ^#^	0.75 ± 0.01^ϕϕϕϕ^, ^####^	0.69 ± 0.01****, ^####^	0.76 ± 0.01^ϕ^, ^####^
	4	0.76 ± 0.01	0.79 ± 0.01^ϕϕϕϕ^, *	0.77 ± 0.01^ϕϕϕϕ^	0.75 ± 0.01^ϕϕϕϕ^, ^##^	0.68 ± 0.01****, ^####^	0.76 ± 0.01^ϕϕ^, ^#^
**Total Power of IBI**	0	8.36 ± 0.93	8.38 ± 0.83	6.35 ± 0.93	8.13 ± 0.70	5.65 ± 0.93*, ^#^	6.77 ± 0.70
	1	12.10 ± 0.69^ϕϕϕ^	9.61 ± 0.62**	9.23 ± 0.69^ϕϕ^, **	9.89 ± 0.52^ϕ^, *	8.54 ± 0.69^ϕϕ^, ***	7.91 ± 0.52****, ^#^, ^∧∧^
	2	12.71 ± 0.89^ϕϕϕ^	9.37 ± 0.79**	11.29 ± 0.89^ϕϕϕϕ^	11.85 ± 0.67^ϕϕϕϕ^, ^#^	9.61 ± 0.89^ϕϕϕ^, *	10.69 ± 0.67^ϕϕϕϕ^
	3	15.75 ± 0.87^ϕϕϕϕ^	9.76 ± 0.78****	11.98 ± 0.87^ϕϕϕϕ^, **	9.22 ± 0.66****	9.66 ± 0.87^ϕϕϕ^, ****	9.43 ± 0.66^ϕϕ^, ****
	4	16.25 ± 0.77^ϕϕϕϕ^	9.15 ± 0.69****	8.22 ± 0.77****	9.28 ± 0.58****	10.20 ± 0.77^ϕϕϕϕ^, ****	9.00 ± 0.58^ϕϕ^, ****

*Ang II, Angiotensin II; LF, low frequency; VLF, very low frequency; HF, high frequency; SBP, systolic blood pressure; IBI, inter-beat-interval.*

*Data was analyzed by Kruskal–Wallis *H* test followed by Tukey’s multiple comparisons test and presented as mean ± SEM.*

*^ϕϕϕϕ^*p* < 0.0001, ^ϕϕϕ^*p* < 0.001, ^ϕϕ^*p* < 0.01, ^ϕ^*p* < 0.05 vs. Baseline.*

****p < 0.0001, ***p < 0.001, **p < 0.01, *p < 0.05 Control vs. all other groups.

*^####^*p* < 0.0001, ^###^*p* < 0.001, ^##^*p* < 0.01, ^#^*p* < 0.05 Ang II vs. NaHS-treated groups.*

*^∧∧∧∧^*p* < 0.0001, ^∧∧∧^*p* < 0.001, ^∧∧^*p* < 0.01, ^∧^*p* < 0.05 30 nmol/h NaHS/Ang II vs. 60 nmol/h NaHS/Ang II.*

Spectral analysis of HR variability showed that VLF_*IBI*_ significantly increased with time in the control but not in the Ang II group (Table 2), suggesting that Ang II attenuated the time-dependent increase in this variable. Co-infusion of NaHS with Ang II partially corrected the Ang II-induced changes in VLF_*IBI*_ at W2 in the 30 nmol/h NaHS/Ang II group and at W3 in the 60 nmol/h NaHS/Ang II group (Figure 2B). However, at W4, VLF_*IBI*_ of all groups was significantly lower than control. Similarly, LF_*IBI*_ increased with time in the control group but not in the Ang II group (Table 2 and Figure 2C). Co-infusion of NaHS with Ang II partially corrected the Ang II-induced changes in LF_*IBI*_ but only at W2 in the 30 nmol/h NaHS/Ang II group, while LF_*IBI*_ was significantly lower at W3 in the 60 nmol/h NaHS/Ang II group (Figure 2C). HF_*IBI*_ was significantly increased in all groups with time except in the Ang II group (Table 2). However, at W4, the control group showed significantly higher HF_*IBI*_ compared with all groups, and no significant differences were found between the Ang II and NaHS/Ang II groups (Table 2 and Figure 2D). A decrease in LF/HF_*IBI*_, an index of cardiac autonomic modulation, was also observed in the Ang II and 30 nmol/h NaHS/Ang II group, with no change in 60 nmol/h NaHS/Ang II group (Table 2 and Figure 2G). TP_*IBI*_ was significantly increased in the control, 60 nmol/h NaHS control, and 60 nmol/h NaHS/Ang II groups with time, whereas this variable was not significantly altered with Ang II (Table 2). Analysis of normalized values shows that both the Ang II infusion alone and co-infusions of ICV NaHS with Ang II significantly reduced nLF_*IBI*_ (Figure 2E) and increased nHF_*IBI*_ (Figure 2F) with time. The nLF_*IBI*_ and nHF_*IBI*_ and LF/HF_*IBI*_ remained unchanged in the control and 60 nmol/h NaHS control group, while 30 nmol/h NaHS control group produced a significantly reduced nLF_*IBI*_ and increased nHF_*IBI*_ with time, resulting in decreased LF/HF_*IBI*_ in this group (Table 2). These data suggest that ICV NaHS can affect Ang II-dependent changes in autonomic variables.

### Central Effects of NaHS on Angiotensin II–Induced Elevation in Iba1^+^ Cells in the PVN

**FIGURE 3:**
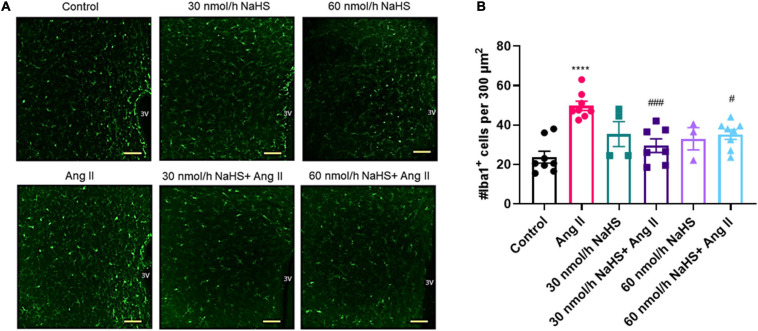
Chronic intracerebroventricular infusion of NaHS reduces angiotensin (Ang) II–induced elevation in ionized calcium binding adaptor molecule 1–positive (Iba1^+^) cells in the paraventricular nucleus (PVN) of Sprague–Dawley rats. **(A)** Representative images of Iba1^+^ cells in the PVN in control, Ang II-treated, and NaHS/Ang II-treated rats (magnification: ×20, scale bar = 100 μm, –0.72 mm to –1.92 mm from Bregma, Paxinos and Watson Rat Brain Atlas). **(B)** Quantification of Iba1^+^ cells per 300 μm^2^ in the PVN of control (*n* = 5), Ang II (*n* = 4), 30 nmol/h NaHS (*n* = 3), 60 nmol/h NaHS (*n* = 2), 30 nmol/h NaHS/Ang II (*n* = 3), and 60 nmol/h NaHS/Ang II (*n* = 4). ****p < 0.0001, Ang II vs. control. ^###^*p* < 0.001, ^#^*p* < 0.05 Ang II vs. NaHS/Ang II-treated rats. Data are presented as the mean ± SEM, one-way ANOVA, Dunn’s multiple comparisons test.

As NaHS is an anti-inflammatory agent (Li et al., 2008; Sidhapuriwala et al., 2009; Aslami et al., 2013), and as microglial cell numbers are reportedly increased in the PVN in rodent Ang II HTN (Shi et al., 2010; Shen et al., 2015), we investigated if ICV NaHS administration would reduce the numbers of Iba1^+^ cells in the PVN in rodent Ang II HTN. We observed a significant increase in the number of Iba1^+^ cells in the PVN of Ang II-infused rats (per 300 μm^2^ area of PVN) compared with the control group (Figures 3A,B: Ang II: 49.6 ± 1.9 vs. control: 23.7 ± 2.3, *p* < 0.0001). Co-infusion of both 30 and 60 nmol/h NaHS resulted in significantly less Iba1^+^ cells compared with the Ang II group (Figures 3A,B: Ang II: 49.6 ± 1.9 vs. 30 nmol/h NaHS/Ang II: 28.5 ± 2.7, *p* < 0.001; vs. 60 nmol/h NaHS/Ang II: 34.5 ± 2.0, *p* < 0.05). These data suggest that ICV NaHS can attenuate Ang II-induced increase in microglial cells in the PVN of rats.

### Alterations in Cecal Microbial Composition in Rodents With Angiotensin II HTN and After Central Administration of NaHS

**FIGURE 4:**
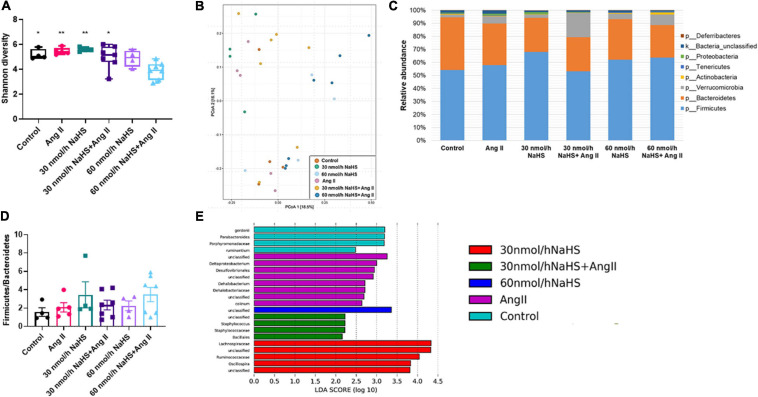
Alterations in cecal microbial composition of Sprague–Dawley rats with angiotensin (Ang) II hypertension and after intracerebroventricular NaHS administration. **(A)** Gut bacterial alpha diversity (Shannon index). Data were analyzed by one-way ANOVA, Tukey’s multiple comparisons test, and presented as minimum to maximum with a median. *p < 0.05, **p < 0.01 vs. 60 nmol/h NaHS/Ang II. **(B)** Gut bacterial beta diversity (principal coordinate analysis, PCoA) determined by permutational analysis of variance (adonis R function or PERMANOVA). Operational taxonomical unit (OTU) abundance is summarized into Bray–Curtis dissimilarities and performed a PCoA ordination. **(C)** Mean relative abundance of gut bacterial phyla identified in cecal samples in each experimental group. **(D)** Ratio of *Firmicutes* to *Bacteroidetes*. Data were analyzed by one-way ANOVA followed by Kruskal–Wallis test and presented as mean ± SEM. **(E)** Linear discriminant analysis effect size (LEfSe) analysis showing enriched taxa in each group. Control (*n* = 4), Ang II (*n* = 5), NaHS (*n* = 4), and NaHS/Ang II-treated rats (*n* = 7).

Considering the association between gut dysbiosis and rodent and human HTN (Yang et al., 2015; Adnan et al., 2017; Li et al., 2017), we investigated the abundance and diversity of gut microbial species in all groups. Alpha diversity (Shannon index) is a measure of richness (how many OTUs) and evenness (how evenly distributed these OTUS are) in a sample (Willis, 2019). We observed no significant differences in alpha diversity (Shannon index) in any of the treatment groups except in the 60 nmol/h NaHS/Ang II group when compared with all other groups but not the 60 nmol/h NaHS group (Figure 4A: 60 nmol/h NaHS/Ang II: 3.9 ± 0.3 vs. control: 5.2 ± 0.2, *p* < 0.05; vs. 30 nmol/h NaHS: 5.6 ± 0.1, *p* < 0.01; vs. Ang II: 5.5 ± 0.1, *p* < 0.01; vs. 30 nmol/h NaHS/Ang II: 5.0 ± 0.4, *p* < 0.05). This suggested a difference in richness (number of taxa) and/or evenness (the relative abundances of those taxa) in cecal fecal bacterial samples from the 60 nmol/h NaHS/Ang II group, and to a certain extent the 60 nmol/h NaHS group compared with all other groups. However, PCoA analysis (Figure 4B) showed no apparent clustering between the groups. In addition, *Firmicutes*, *Bacteroidetes*, and *Verrucomicrobia* were the most abundant bacterial phyla in all groups; however, abundance in these phyla was not significantly different between the groups (Figure 4C). In addition, we observed no difference in the F/B ratios between the treatment groups (Figure 4D).

### Central Administration of NaHS Diminished Angiotensin II Effects on the Abundance of Cecal *Desulfovibrionales*, *Deltaproteobacterium*, and *Dehalobacterium*

LEfSe analysis of specific bacterial taxa demonstrated a significant increase in the abundance of, among others, *Desulfovibrionales*, *Deltaproteobacterium*, and *Dehalobacterium* in the Ang II group (in purple) compared with all other groups (Figure 4E). The observed increase in abundance of sulfate-reducing *Desulfovibrionales* after Ang II infusion suggested that the production of bacterial derived H_2_S may be altered in the Ang II group, and that this can be corrected with ICV NaHS treatment. In addition, we observed higher abundance of *Parabacteroides* in the control group (in blue) compared with all other groups, higher abundance of *Staphylococcus* and *Bacillales* in the 30 nmol/h NaHS/Ang II group (in green), and *Ruminococcaceae*, among others, in the 30 nmol/h NaHS group (in red) compared with all other groups including the control group (in blue, Figure 4E).

### Effect of Angiotensin II HTN and Central Administration of NaHS on Circulating H_2_S Levels

**FIGURE 5:**
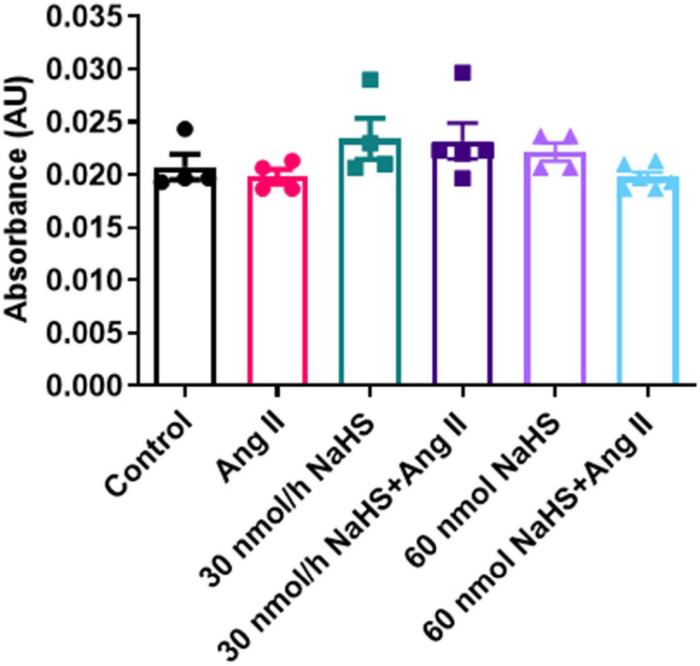
Hydrogen sulfide levels in plasma of Sprague–Dawley rats with angiotensin (Ang) II hypertension and after intracerebroventricular NaHS administration as measured by methylene blue method. Data were analyzed by one-way ANOVA followed by Kruskal–Wallis test and presented as mean ± SEM. AU: arbitrary unit. Control (*n* = 4), Ang II (*n* = 5), NaHS (*n* = 4), and NaHS/Ang II-treated rats (*n* = 7).

Considering the increase in the sulfate-reducing *Desulfovibrionales* in the Ang II group (Figure 4E) and a significant decrease in Shannon (alpha) cecal bacterial diversity in the 60 nmol/h NaHS/Ang II group (Figure 4A), we measured circulating H_2_S levels in all rats at endpoint (W4) using methylene blue method (Shen et al., 2011; Donertas Ayaz and Zubcevic, 2020). We observed no difference in circulating H_2_S levels in any of the treatment groups (Figure 5). These data suggest that rodent HTN induced by systemic Ang II infusion (200 ng/kg/min) may be independent of any changes in circulating H_2_S. In addition, ICV infusion of NaHS can reduce Ang II-dependent increase in sulfate-reducing cecal *Desulfovibrionales* with no effect on circulating H_2_S.

## Discussion

Our data reveal the following findings: (1) Central administration of NaHS, an H_2_S donor, attenuated Ang II-induced increase in BP in a dose-dependent manner. This may be in part due to improvement in the autonomic regulation of BP, largely reflecting in effects on the sympathetic vasomotor drive derived from spectral analysis of SBP waveform signal. (2) Central administration of NaHS alleviated Ang II-dependent increase in the Iba1^+^ cells in the PVN. These data provide further support for a role of central H_2_S in regulation of cardiovascular function (Ufnal et al., 2008; Sikora et al., 2014; Duan et al., 2015; Yu et al., 2015; Liang et al., 2017). (3) Ang II infusion increased the abundance of select gut bacteria, which was prevented by ICV co-administration of NaHS. As we observed no change in circulating H_2_S in any of the treatment groups, this suggests that the anti-hypertensive effects of H_2_S are primarily mediated via the CNS in the current study.

Both the gut bacteria and their host reportedly produce H_2_S (Kimura, 2014; Donertas Ayaz and Zubcevic, 2020) and gut-derived H_2_S may contribute to the regulation of BP (Tomasova et al., 2016; Huc et al., 2018). In line with the latter, we have recently shown a positive correlation between the gut bacterial and circulating H_2_S levels in the spontaneously hypertensive rat (Donertas Ayaz and Zubcevic, 2020). Indeed, host microbiota may play an important role in controlling H_2_S bioavailability and metabolism (Shen et al., 2013) and gut dysbiosis can have an effect on the plasma H_2_S (Weber et al., 2016). As reduced systemic H_2_S levels are reported in rodent and human HTN (Chen et al., 2007; Xiao et al., 2018; Donertas Ayaz and Zubcevic, 2020), we also investigated circulating H_2_S levels in Ang II-induced rodent HTN. We show no association between Ang II rodent HTN and plasma H_2_S in the current experimental paradigm. Interestingly, we did observe an increased abundance of sulfate-reducing *Desulfovibrio* in rats with Ang II HTN. It has previously been shown that increased H_2_S production in the gut can lead to localized gastrointestinal inflammation (Singh and Lin, 2015), and Ang II is also implicated in inflammation (Benigni et al., 2010; Santisteban et al., 2015; Zubcevic et al., 2017; Satou et al., 2018). Although we did not measure fecal H_2_S, unchanged circulating H_2_S in Ang II-infused rats suggests that the H_2_S effects observed in the current study were not mediated via systemic H_2_S. From this, the anti-hypertensive effects of ICV infusion of NaHS appear to be primarily mediated via the CNS, as increasing the availability of central H_2_S modified cardiovascular responses without affecting the systemic H_2_S levels. It is noteworthy that the current study used methylene blue method for detection of H_2_S in plasma (Zhuo et al., 2009; Donertas Ayaz and Zubcevic, 2020). Future studies should employ analytical techniques such as gas/ion chromatography, HPLC, and polarographic electrodes among others to increase sensitivity of H_2_S detection in gut and plasma (Kolluru et al., 2013).

Ang II in the CNS modulates neurohumoral pathways involved in autonomic control of BP. Ang II receptor activation within the PVN is a major contributor to chronic sympatho-excitation, while Ang II in the nucleus of the solitary tract reduces the parasympathetic control of the heart (Paton and Raizada, 2010). Thus, one goal of the current study was to evaluate the effects of chronic, concomitant ICV administration of NaHS on autonomic effects exerted by Ang II. Our findings show that Ang II infusion increased LF_*SBP*_, a marker of sympathetic vasomotor modulation (deBoer et al., 1987; Madwed et al., 1989), which is consistent with reports in other rodent models of HTN (Waki et al., 2006; Zubcevic et al., 2014, 2017; Chaar et al., 2016). Importantly, we show that ICV NaHS co-infusion attenuated the Ang II effects on LF_*SBP*_. We used established stereotaxic coordinates for ICV infusion (Li et al., 2006; Jun et al., 2012; Buttler et al., 2017). However, we acknowledge that ICV administration may deliver NaHS to ventricular brain structures other than PVN (Hashimoto et al., 2005; Buttler et al., 2017). Thus, it is possible that effects of NaHS may be exerted via action on several brain nuclei in the current study. We also investigated the effects of our treatments on VLF, LF, and HF of IBI as indices of autonomic modulation of HR. The VLF_*IBI*_ variable is reportedly related to changes in the renin–angiotensin system and thermoregulation (Taylor et al., 1998; Pumprla et al., 2002) as well as the parasympathetic modulation (Taylor et al., 1998). The HF_*IBI*_ is considered an index of cardiac vagal efferent modulation, while LF_*IBI*_ reportedly contains both vagal and cardiac sympathetic components (Shaffer and Ginsberg, 2017). Thus, any changes in these variables are likely to be reflected in modified autonomic input to the heart and potentially changes in BP. However, while the effects of Ang II on these variables are significant, the effect of ICV NaHS treatment on Ang II-induced changes on VLF and LF of IBI is only partial and sporadic, with no significant effect on HF of IBI. Moreover, the effects of NaHS infusion alone on both VLF and LF of IBI appear to be significant at select time points and doses of NaHS, suggesting NaHS alone may have neuromodulatory effects in control conditions. This may potentially be due to effects of H_2_S on astrocytes (Nagai et al., 2004; Lu et al., 2008) and/or neurons (Han et al., 2005; Umemura and Kimura, 2007; Garcia-Bereguiain et al., 2008) as changes in calcium currents and effects on GABA and glutamate neurotransmission have previously been observed in astrocytes and neurons, respectively in response to central H_2_S. However, we did not investigate these in the current study. Ang II did significantly alter nLF_*IBI*_ and nHF_*IBI*_, which are derived by normalizing the raw values of LF_*IBI*_ and HF_*IBI*_, respectively, to the value of TP_*IBI*_, and ICV NaHS co-treatment appeared to have corrected this effect of Ang II on these two variables. Moreover, LF/HF_*IBI*_, an index of cardiac autonomic balance, significantly decreased in the Ang II group, and 60 nmol/h ICV NaHS co-treatment partially corrected this decrease. However, interpretation of these changes in IBI variables are challenging due to the reported participation of parasympathetic modulation in both LF and HF components (Billman, 2013). Thus, these results suggest that central H_2_S may counteract the effects of Ang II on autonomic function via effects on pre-autonomic neurons that regulate BP.

Interestingly, we observed an increase in the IBI variables including HF_*IBI*_ over time in the control group (Table 2), which aligns with the time-dependent decrease in HR in the same group. We have previously shown that HR can decrease with age in normotensive rodents (Zubcevic et al., 2009). Thus, attenuation of these time-dependent changes in HF_*IBI*_ and HR in the Ang II group suggests deregulation of cardiac parasympathetic drive after Ang II infusion, as it has previously been reported (Miller and Arnold, 2019; Dorey et al., 2020; Sharma et al., 2021). However, this effect of Ang II on HF_*IBI*_ was not significantly affected by ICV NaHS co-treatments, suggesting lesser effects of central infusion of NaHS on parasympathetic control of BP in our study. Further studies are needed to fully understand the effects of central NaHS on cardiac autonomic control.

Left ventricular hypertrophy is a secondary manifestation of HTN resulting from adaptation of heart muscle to accommodate the increased cardiac work via the compensatory hypertrophic response (Li et al., 2019a). In line with our BP results, we observed increased LV wall thickness in Ang II-infused group compared with control, which was significantly attenuated by chronic ICV 60 nmol/h but not the 30 nmol/h NaHS treatment. However, heart weight analysis showed no significant differences between the groups in the current study. Thus, the observed changes in wall thickness may be due to hypertrophy and/or dilation effects. However, we did not perform heart fibrosis and diameter analyses in the current study. Further studies are needed to determine dose-dependent long-term cardiac effects of NaHS in HTN.

Considering the reported anti-inflammatory properties of H_2_S, we investigated whether central administration of NaHS can attenuate the previously reported increase in microglial cells in the PVN, a recognized hallmark of rodent HTN (Shi et al., 2010; Shen et al., 2015). Microglial cells in the CNS express Iba1, a microglia/macrophage-specific calcium-binding protein (Sasaki et al., 2001), which is involved in the membrane ruffling processes of macrophages/microglia and confers the motile properties of these cells (Kanazawa et al., 2002). As previously reported, Ang II infusion significantly increased the number of PVN microglial cells (Shi et al., 2010; Santisteban et al., 2015; Sharma et al., 2019). Here, we report that central NaHS treatment reduced Iba1^+^ cells in the PVN of Ang II-infused rats. Although we did not investigate microglial morphology in the current study, others have previously shown that both NaHS and H_2_S can inhibit microglial activation *in vitro* (Hu et al., 2007; Zhang et al., 2014) and *in vivo* (Zhang et al., 2014; Kumar and Sandhir, 2019; Kumar et al., 2020). It is proposed that Ang II can stimulate the production of pro-inflammatory cytokines and reactive oxygen species in both neurons and microglia (Kang et al., 2009; Shi et al., 2010; Jun et al., 2012; Qi et al., 2013; Shen et al., 2015). This may affect neuronal activity and contribute to the increase in the sympathetic outflow and BP in rodent HTN (Kang et al., 2009; Shi et al., 2010; Jun et al., 2012; Qi et al., 2013; Shen et al., 2015). Liang et al. (2017) have previously shown that chronic PVN infusion of a separate H_2_S donor, GYY4137, decreased BP and plasma noradrenaline levels in rodent salt HTN while simultaneously reducing reactive oxygen species and IL-1β in the PVN. However, H_2_S may also have direct neuronal effects (Szabo, 2021), warranting further investigation of H_2_S in regulation of activity of pre-sympathetic neurons (Guo et al., 2011; Centurion et al., 2016; Dominguez-Rodriguez et al., 2017).

An increase in the F/B ratio, a marker of generalized gut dysbiosis, has been reported in several rodent models of HTN (Yang et al., 2015; Durgan et al., 2016; Marques et al., 2017; Chen et al., 2019; Hsu et al., 2020; Robles-Vera et al., 2020) and humans with HTN (Yang et al., 2015; Silveira-Nunes et al., 2020). However, others have reported no change in the F/B ratio in select animal models of HTN (Mell et al., 2015; Galla et al., 2018; Cheema and Pluznick, 2019). In line with the latter, we did not observe a significant change in the F/B ratios in the current study. However, Ang II did produce a significant shift in the abundance of specific bacteria including but not limited to *Desulfovibrio*, *Deltaproteobacterium*, and *Dehalobacterium*. The genus *Desulfovibrio* in the *Deltaproteobacteria* class is one of the most abundant sulfate-reducing bacteria (SRB) in the human colon (Dordevic et al., 2021). Higher abundance of genus *Desulfovibrio* observed in the Ang II group was in agreement with the previous reports of other hypertensive rodent models and human patients (Adnan et al., 2017; Li et al., 2017; Dan et al., 2019; Thomaz et al., 2021). *Desulfovibrio* abundance is also positively correlated with Ang II levels in rats with high-carbohydrate, high-fat diet–induced HTN (Thomaz et al., 2021). These and our findings further support the possible involvement of SRB in HTN. H_2_S produced by gut bacteria is an important regulator of gut health, and high concentrations of H_2_S are involved in gut inflammation (Dordevic et al., 2021). Thus, reducing gut *Desulfovibrio*, as we have shown in the current study, may be beneficial in regulation of BP. Thus, while CNS H_2_S may be beneficial, higher abundance of SRB may be associated with HTN. However, a cause–effect relationship between SRB and HTN is not clear. On one hand, studies suggest H_2_S involvement in gut inflammation (Dordevic et al., 2021), and on the other, H_2_S may be protective of the mucosal integrity in the gut (Wallace et al., 2018). Further studies employing genomic sequencing of SRB and investigation of fecal and host H_2_S levels in rodents and humans with HTN are needed to clarify this matter.

To the best of our knowledge, no study to date has reported an association between *Dehalobacterium* and HTN, but a positive correlation between the abundance of *Dehalobacterium* and obesity and higher body mass index, which are also associated with HTN (Jones et al., 1994; Wilson et al., 2002; Zhang et al., 2019), has been previously reported (Fu et al., 2015; Thomaz et al., 2021). Here, we observe that central administration of NaHS normalized the levels of *Desulfovibrio*, *Deltaproteobacteria*, and *Dehalobacterium* in Ang II-infused rats. These bacterial shifts may be independent of systemic Ang II effects, as systemic Ang II levels remain constant in all experimental groups. We further observed a decrease in *Parabacteroides* and *Porphyromonadaceae* in the Ang II group, but as these also remained less abundant in all other treatment groups, they may not be associated with regulation of BP. Further studies are needed to elucidate the precise mechanisms of host–microbiota interactions and how they may be involved in hypertensive rodent phenotype and generation of H_2_S.

Gender-associated differences in BP have been observed in animals and in humans (Reckelhoff, 2001; Lim et al., 2002; Mishra et al., 2016). Thus, one limitation of the current study is the exclusion of female rats, as the potential of sex differences in the responses to NaHS and Ang II may be important. In addition, sex-dependent gut bacterial differences have been reported (Jasarevic et al., 2016; Beale et al., 2019). Future experiments should investigate potential sex-dependent responses to H_2_S and NaHS treatment in relation to regulation of BP and HTN.

In conclusion, our present data show that central administration of an H_2_S donor, NaHS, attenuates BP increase and improves autonomic function in Ang II HTN independently of circulating H_2_S, while also decreasing SRB in the gut despite continuous systemic Ang II infusion. Moreover, centrally administered NaHS attenuated the Ang II-induced increase in microglial cells in the PVN. Thus, H_2_S is a potential therapeutic target for neurogenic HTN and the underlying mechanisms of central H_2_S effects in HTN warrant future investigation.

## Data Availability Statement

The original contributions presented in the study are included in the article/Supplementary Material, further inquiries can be directed to the corresponding author/s.

## Ethics Statement

The animal study was reviewed and approved by University of Florida Institutional Animal Care and Use Committee.

## Author Contributions

BDA designed the study, carried out the research, analyzed and interpreted the results, and wrote the manuscript. ACO contributed to the analysis and interpretation of data and drafting of the manuscript. WLM, TR, AMA, and RKS contributed to data acquisition. BS participated in the design of the study and edited the manuscript. JZ contributed to the design of the study, access to research components, interpretation of the results, and drafting and editing of the manuscript. All authors read and approved the final manuscript version.

## Conflict of Interest

The authors declare that the research was conducted in the absence of any commercial or financial relationships that could be construed as a potential conflict of interest.

## Publisher’s Note

All claims expressed in this article are solely those of the authors and do not necessarily represent those of their affiliated organizations, or those of the publisher, the editors and the reviewers. Any product that may be evaluated in this article, or claim that may be made by its manufacturer, is not guaranteed or endorsed by the publisher.

## Footnotes

1
https://huttenhower.sph.harvard.edu/galaxy

## Supplementary Material

Supplementary Material Bacterial sequencing ICV NaHS Ang II hypertension.
